# Prediction of primary venous thromboembolism based on clinical and genetic factors within the U.K. Biobank

**DOI:** 10.1038/s41598-021-00796-4

**Published:** 2021-11-01

**Authors:** David A. Kolin, Scott Kulm, Olivier Elemento

**Affiliations:** 1 Caryl and Israel Englander Institute for Precision Medicine, Weill Cornell Medicine, New York, NY USA; 2 Physiology, Biophysics, and Systems Biology, Weill Cornell Medicine, New York, NY USA

**Keywords:** Genetics, Risk factors, Epidemiology, Genetics research

## Abstract

Both clinical and genetic factors drive the risk of venous thromboembolism. However, whether clinically recorded risk factors and genetic variants can be combined into a clinically applicable predictive score remains unknown. Using Cox proportional-hazard models, we analyzed the association of risk factors with the likelihood of venous thromboembolism in U.K. Biobank, a large prospective cohort. We then created a polygenic risk score of 36 single nucleotide polymorphisms and a clinical score determined by age, sex, body mass index, previous cancer diagnosis, smoking status, and fracture in the last 5 years. Participants were at significantly increased risk of venous thromboembolism if they were at high clinical risk (subhazard ratio, 4.37 [95% CI, 3.85–4.97]) or high genetic risk (subhazard ratio, 3.02 [95% CI, 2.63–3.47]) relative to participants at low clinical or genetic risk, respectively. The combined model, consisting of clinical and genetic components, was significantly better than either the clinical or the genetic model alone (*P* < 0.001). Participants at high risk in the combined score had nearly an eightfold increased risk of venous thromboembolism relative to participants at low risk (subhazard ratio, 7.51 [95% CI, 6.28–8.98]). This risk score can be used to guide decisions regarding venous thromboembolism prophylaxis, although external validation is needed.

## Introduction

Both clinical and genetic factors drive the likelihood of venous thromboembolism, the leading cause of preventable hospital deaths^1^. Considerable evidence demonstrates that individuals exposed to clinical risk factors, such as cancer, oral contraceptive pills, high body mass index, recent hospitalization, and major surgery, have markedly increased risk of venous thromboembolism^2–7^.

While clinical factors account for a significant proportion of thromboembolic risk, over 60% of variation in the risk of venous thromboembolism can be attributed to genetic factors^8^. Factor V Leiden and Factor II Mutation, two monogenic variants, were first described in the 1990s^9–14^. Since then, genome-wide association studies have identified over 20 additional loci. Risk alleles, when combined into a polygenic score, are capable of quantifying genetic susceptibility and are often more effective at predicting risk than rare monogenic variants alone^15–22^.

Currently, understanding of clinical and genetic risk for venous thromboembolism guides both prophylaxis and treatment^23–26^. Models that predict venous thromboembolism do exist, but many such models are designed for specific sub-populations^27,28^. A continuous score for prognosticating venous thromboembolism—by combining clinical and genetic factors—is not routinely used for the general population. In order to address these gaps, we first explored clinical risk factors for venous thromboembolism in U.K. Biobank, a large longitudinal cohort. We next transitioned from causal modeling to predictive modeling, ultimately developing and validating a multivariable venous thromboembolism risk model, comprised of clinical and genetic predictors.

## Methods

### Study population and design

The U.K. Biobank is a prospective cohort of 502,536 participants between the ages of 40 and 69 years recruited from the United Kingdom from 2006 to 2010. Baseline information was gathered during an in-person interview, and in-patient venous thromboembolism events were collected prospectively for all hospital episodes. Specifically, all risk factors were measured at baseline. Because the vast majority of participants in the U.K. Biobank are white, for predictive modeling, the U.K. Biobank was subset to white, ethnically British individuals meeting genetic quality controls, as described previously^21,29,30^. While inclusion of all individuals would be preferred, this downsizing was necessary to reduce confounding from ancestral groups. A training set, used to derive the score, and a validation set, used to quantify effect estimates were partitioned from the subset population. The training set was derived from the phase 1 release of the U.K. Biobank (n = 126,247), and the validation set was derived from the phase 2 release of the U.K. Biobank (n = 271,459), as described previously^21^. Differences between the training and testing data were minimal. Written informed consent was obtained for all participants in U.K. Biobank.

### Study outcome

Following the example of previous work, we defined venous thromboembolism as pulmonary embolism or deep vein thrombosis^4^. Additional details regarding the coding of venous thromboembolic events are provided in eTable 1 in Supplement 1. Because we were interested in the first occurrence of venous thromboembolism, participants with a history of venous thromboembolism, occurring before baseline, were excluded from analyses. Participants were considered at risk for venous thromboembolism at baseline and were censored at death, loss to follow-up, or the last date of follow-up (March 31, 2017 for England, October 31, 2016 for Scotland, and February 29, 2016 for Wales).

### Polygenic risk scores

We constructed a polygenic risk score from 36 single nucleotide polymorphisms (SNPs), all with genome-wide significance in previously published genome wide association studies of venous thromboembolism^31–37^. These 36 SNPs were chosen from a total possible set of 102 SNPs through elastic net logistic regression. The genotypic input to this regression was generated by first organizing summary statistics of significant SNPs to venous thromboembolism, removing duplicate or ambiguous SNPs, flipping the effect to match strands, and counting the number of occurrences of the effect allele for each U.K. Biobank participant. Under five-fold cross validation, the optimal lambda value for the regression was determined within the training set of data. A regression model was then fit upon the full training set to determine the final effect values for each SNP. The product of the final effect value and the count of effect alleles summed over all SNPs, in both training and validation sets, generated the polygenic risk scores. Missing genotypes were imputed to the population’s allele frequency. Details of the included studies and allele effect value are provided in eTable 2 in Supplement 1.

In addition to the novel polygenic risk score, a score described by *De Haan *et al*.* was also produced for later comparison to our own score^38^. The genotypic information for each listed variant was processed under the same steps as the self-derived score. Of the total 31 SNPs listed in the publication, 27 were available in the U.K. Biobank for scoring. The effect allele was determined by a simple logistic regression of the number of major alleles against venous thromboembolism status. The final De Haan score was then created by summing the product of the count of major alleles and the effect values utilized within the *De Haan *et al. publication.

### Statistical analysis

The statistical analysis was completed in two parts. In the first section of the statistical analysis, we investigated the association of clinical risk factors with venous thromboembolism using a causal modeling approach on the full dataset. In the second section of the statistical analysis, we developed and validated a model of clinical and genetic factors to predict venous thromboembolism using a predictive modeling approach.

First, the association of nine established risk factors, ten of the most common medications, and ten non-cancer illnesses with incident venous thromboembolism was assessed using Cox proportional-hazard models. Models were adjusted for age, sex, body mass index (BMI), previous cancer diagnosis, smoking status, alcohol intake frequency, use of oral contraceptive pills, use of hormone replacement therapy, fracture in the last 5 years, and the first four principal components of ancestry. The principal components were included, as they control confounding by ancestry, an approach commonly taken in other investigations^21^.

Second, a clinical risk score, a genetic risk score (based on polygenic risk), and a combined score were used to predict incident venous thromboembolism. Based on an evaluation of the training dataset, the clinical risk score was created on an eight-point scale score using six risk factors for venous thromboembolism: sex, age, BMI, smoking status, fracture in the last 5 years, and previous cancer diagnosis (eTable 3 in Supplement 1). There was minimal missing data in the dataset, but participants with missing data were imputed to the mode. After creating a genetic risk score based on polygenic risk, the clinical and genetic scores were combined into a single score by adding both scores proportional to their subhazard ratios, derived from the training set.

The genetic and combined score were then used to categorize study participants into three risk categories: low risk (lowest two deciles), intermediate risk (deciles three to eight), and high risk (top two deciles). The discrete eight point clinical score led to approximate decile categorizations as follows: low risk (0–2 points), intermediate risk (3–4 points), and high risk (5–8 points). The genetic and clinical scores served as the two primary predictors within the Fine-Gray model, fit upon the training set and assessed on the testing set. Subhazard ratios and concordance values were directly extracted from the fit model, and cumulative incidence predictions were computed with the *survfit* function. The primary predictors were used to establish risk groups according to the following definitions: low (1st quintile), intermediate (2–4th quintile), high (5th quintile). The fit of each model was measured by using concordance for Fine-Gray models and area under the curve (AUC) for logistic regression models. Additional nested models were fit with and compared with analysis of variance (ANOVA) tests. All statistical tests were 2-sided. All of the analyses were performed with the use of R software, version 4.0 (R Project for Statistical Computing) and associated packages (eTable 4 in Supplement 1). Statistical results for the predictive modeling portion of this study were reported following the transparent reporting of a multivariable prediction model for individual prognosis or diagnosis (TRIPOD) guidelines.

### Ethical statements

Experimental protocols were approved by the UK Biobank ethics committee. All methods were carried out in accordance with relevant guidelines and regulations.

## Results

### Participant characteristics and established risk factors

There were a total of 4843 venous thromboembolic events amongst the 502,536 participants in U.K. Biobank, leading to an overall incidence rate of 0.96%. The incidence in person-years was 12 cases per 10,000 person-years. The mean age of participants was 56.5 years, 54.4% of participants were female, and the mean BMI was 27.43 [95% CI, 27.42–27.45]. (eTable 5 in Supplement 1). The mean follow-up time was 7.97 years, 0.3% of the total cohort withdrew early, and 4.0% died before the final date of follow-up.

**Figure 1:**
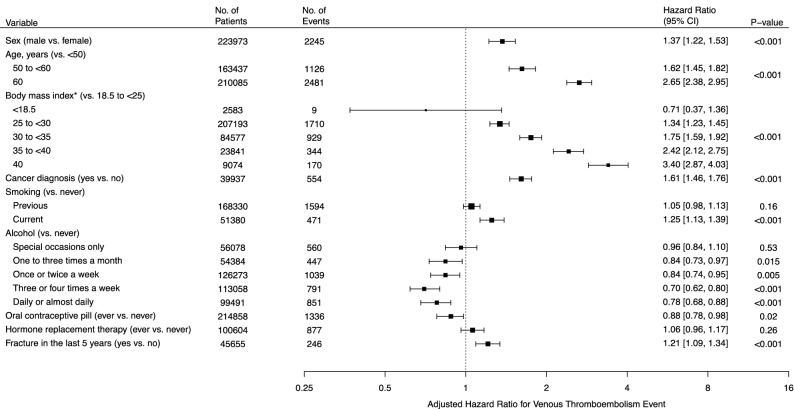
Adjusted hazard ratios for primary venous thromboembolism for established risk factors. Shown are the adjusted hazard ratios for primary venous thromboembolism for nine established risk factors. For any individual risk factor, models were adjusted for all other established risk factors and the first four principal components of ancestry. The I bars represent 95% confidence intervals. Body mass index (*) was measured in kg/m^2^.

We assessed the association of nine known risk factors with venous thromboembolism using data from all participants in the U.K. Biobank. Class 3 obesity (BMI ≥ 40 kg/m^2^), relative to normal weight, was the only risk factor associated with over a threefold increase in the risk of venous thromboembolism (hazard ratio [HR], 3.40 [95% CI, 2.87–4.03]) (Fig. 1). Perhaps surprisingly, participants who had ever used oral contraception had a 12% decreased risk of venous thromboembolism (HR, 0.88 [95% CI, 0.78–0.98]). In exploratory analyses of the length of use of contraception, we found that women who used contraception for at least 20 years were also at decreased risk of venous thromboembolism (HR, 0.80 [95% CI, 0.65–0.98]) (eTable 6 in Supplement 1). However, an insignificant trend of venous thromboembolism risk was observed when length of oral contraceptive use was analyzed on a continuous scale (*P* = 0.24). Further analyses revealed that, of the 4919 current users of oral contraceptive pills, 935 were taking desogestrel (Cerazette 75 µg tablets) (eTable 7 in Supplement 1). Compared to fully adjusted models, univariable risk ratios of death from venous thromboembolism identified similar patterns in risk (eFigure 1 and eTable 8 in Supplement 1).

### Risk of venous thromboembolism with common medications and non-cancer illnesses

**Figure 2:**
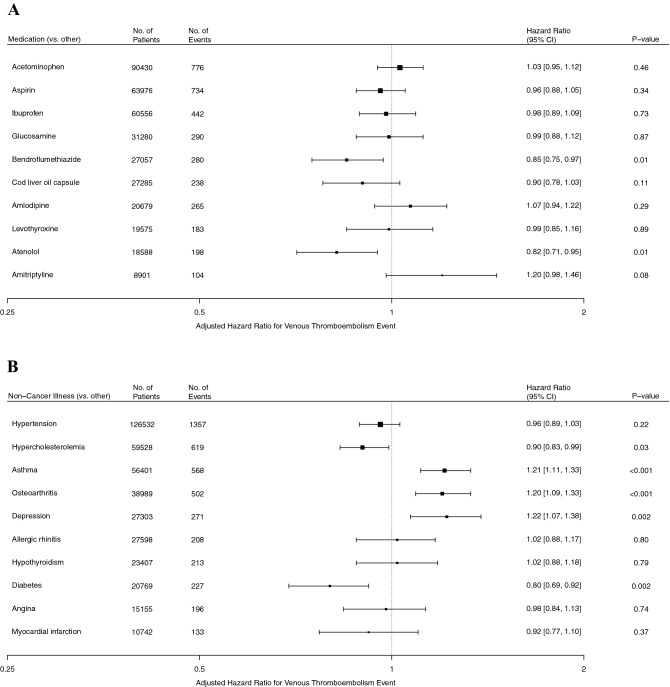
Adjusted hazard ratios for primary venous thromboembolism for common medications and non-cancer illnesses. Shown are the adjusted hazard ratios for primary venous thromboembolic events for two factors of interest: common medications (**A**) and non-cancer illnesses (**B**). All estimates were adjusted for all nine established risk factors and the first four principal components of ancestry. The I bars represent 95% confidence intervals.

In order to better understand the specific clinical risk factors associated with risk of venous thromboembolism, we also analyzed the association of common medications and non-cancer illnesses with venous thromboembolic risk. Of the ten most common medications in U.K. Biobank, two were associated with decreased risk of venous thromboembolism (Fig. 2). Hazard ratios for bendroflumethiazide and atenolol were 0.85 [95% CI, 0.75–0.97] and 0.82 [95% CI, 0.71–0.95], respectively. No other medication was associated with primary venous thromboembolic events.

Participants with asthma, osteoarthritis, and depression were at minimum at 20% increased risk of venous thromboembolism. Hypercholesterolemia was associated with a 10% decreased risk of venous thromboembolism (HR, 0.90 [95% CI, 0.83–0.99]), and diabetes was associated with a 20% decreased risk of venous thromboembolism (HR, 0.80 [95% CI, 0.69–0.92]). However, for hypercholesterolemia and diabetes, evidence of any association was lost after adjusting for common therapies (eTable 9 in Supplement 1). We also analyzed the risk of venous thromboembolism with common cancer subtypes and fracture sites (eFigures 2–3 in Supplement 1). We found that fully and minimally adjusted models of any venous thromboembolism event, which included recurrent events, generated similar results (eFigures 4–13 in Supplement 1).

### Model fitting to determine risk factor importance

We then shifted our focus to predictive modeling, developing and validating Fine-Gray models comprised of genetic, clinical, and combined scores. All models were fit upon the training phase (1131 events amongst 126,247 participants) and assessed on the testing phase (2295 events amongst 269,164 participants). The training and testing sets were similar across a range of baseline factors (eTable 10 in Supplement 1). A total of 4657 participants had missing data in at least one risk factor (eTable 11 in Supplement 1).

**Figure 3:**
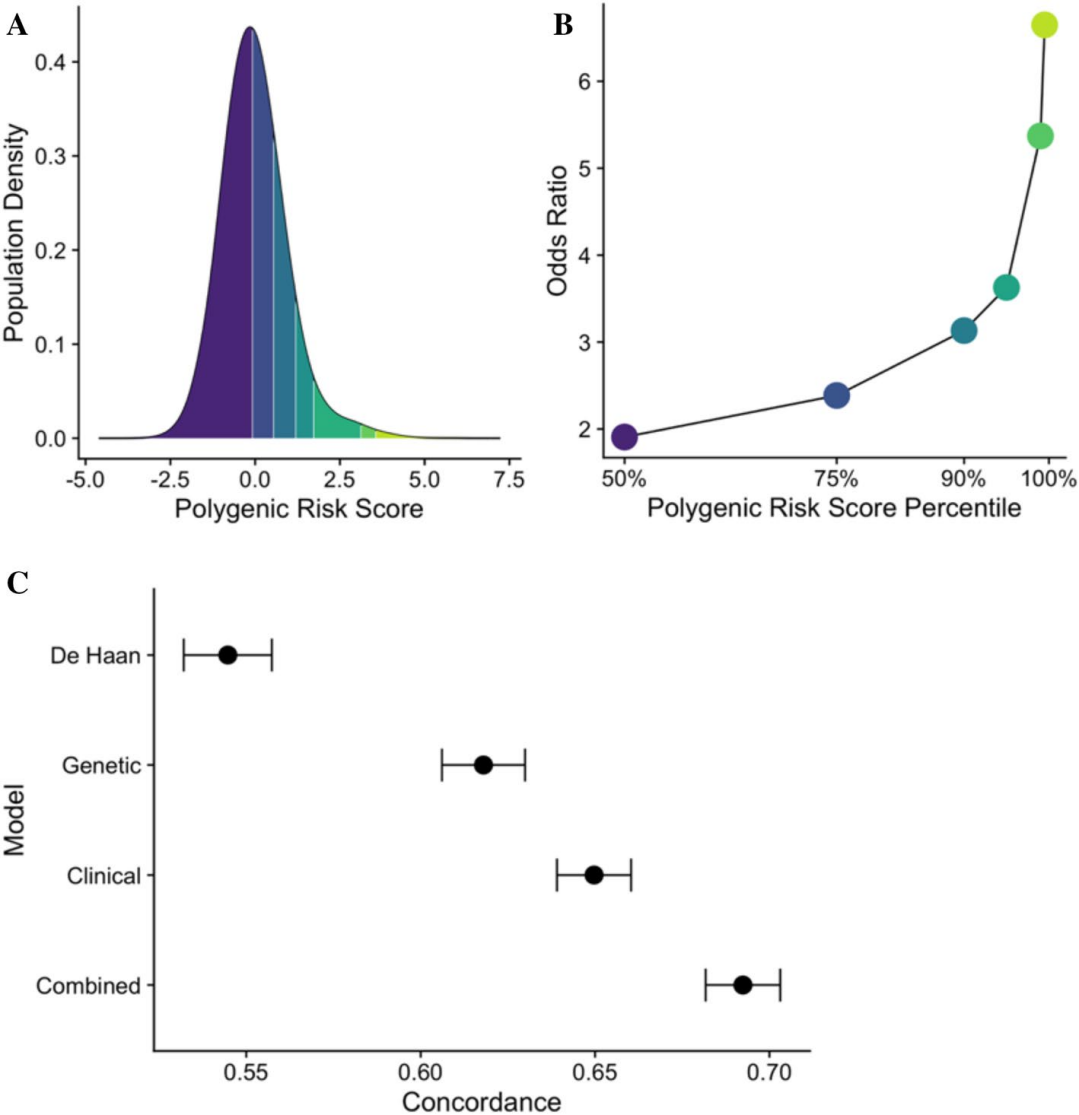
Model performance of a genetic, clinical, and combined score. (**A**) Shows the density of the polygenic risk score stratified by the 50th, 75th, 90th, 95th, 99th and 99.5th percentiles. (**B**) Shows the odds ratio for each polygenic risk score percentile. (**C**) Shows the concordance values, derived from Fine-Gray models, for the De Haan, genetic, clinical, and combined scores. The concordance of the combined model is significantly higher than the concordance of each of the other models (*P* < 0.001). The I bars represent 95% confidence intervals.

The genetic score was predictive of venous thromboembolic events (concordance, 0.62 [95% CI, 0.61–0.63]) (Fig. 3). The odds ratio for participants in the top polygenic risk score percentile compared to the bottom 99 percentiles was 5.37 [95% CI, 5.34–5.40]. Comparatively, the De Haan score generated a lower concordance of 0.54 [95% CI, 0.53–0.55]. A model with the clinical score generated a concordance of 0.65 [95% CI, 0.64–0.65]. The combined score of genetic and clinical factors generated a concordance of 0.69 [95% CI, 0.68–0.70], which was significantly better than either the genetic or the clinical model alone (*P* < 0.001). All model coefficients were extracted for clinical implementation (eTables 12 and 13 in Supplement 1). Additional analyses showed an absence of additional interactions, clustering, residual patterns, or other signs of poor model fit (eFigures 14–20 in Supplement 1).

### Risk stratification

**Figure 4:**
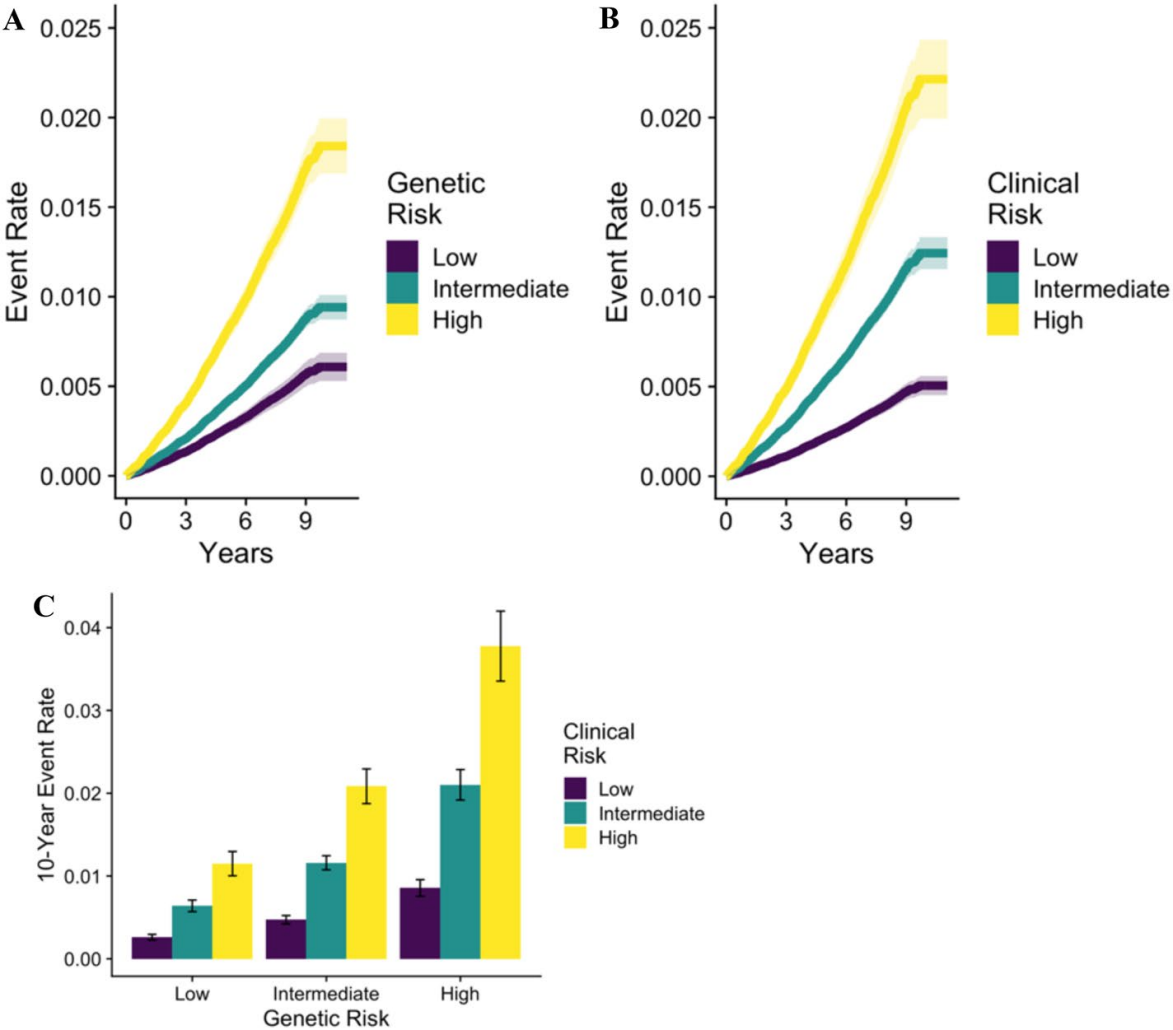
Prediction of venous thromboembolism. (**A**) and (**B**) Show the rates of venous thromboembolism derived from Fine-Gray models for the genetic and clinical score, respectively. (**C**) Shows 10-year event rates for venous thromboembolism, stratified by both the clinical score and genetic score. The I bars represent 95% confidence intervals.

Genetic, clinical, and combined scores were used to stratify risk by comparing participants at low, intermediate, and high risk (Table 1). Participants at high clinical or high genetic risk had 337% (subhazard ratio [SHR], 4.37 [95% CI, 3.85–4.97]) and 202% (SHR, 3.02 [95% CI, 2.63–3.47]) increased risk of venous thromboembolism, respectively, relative to participants at low clinical or low genetic risk. Participants at high clinical and genetic risk had over a threefold (SHR, 3.22 [95% CI, 2.38–4.34]) increased risk of venous thromboembolism relative to participants at high clinical yet low genetic risk, indicating the added benefits of accounting for genetic factors. Alternatively, participants at high genetic risk yet low clinical risk had a 76% (SHR, 0.24 [95% CI, 0.19–0.30]) decreased risk of venous thromboembolism compared to participants at high genetic and high clinical risk, suggesting that clinical factors can attenuate genetic predispositions. Participants in the high risk group of the combined score were at a nearly eightfold (SHR, 7.51 [95% CI, 6.28–8.98]) increased risk of venous thromboembolism relative to participants in the low risk group (eFigure 21 in Supplement 1). When the clinical score and genetic score were analyzed independently, participants at both high clinical and genetic risk, 2.1% of the U.K. Biobank Cohort, had over 12-fold (SHR, 12.21 [95% CI, 8.99–16.59]) greater risk of venous thromboembolism than participants at both low clinical and genetic risk (Fig. 4). The 10-year event rate was 3.77% for participants at high clinical and genetic risk, and 0.26% for participants at low clinical and genetic risk.

**Table 1:** Risk group definitions.

Score	Risk group	Lower limit	Upper limit	No. of participants (%)
Genetic	Low	− 4.01	− 0.81	54,292 (20.0)
	Intermediate	− 0.81	0.71	162,875 (60.0)
	High	0.71	6.63	54,292 (20.0)
Clinical	Low	0	2	106,781 (39.3)
	Intermediate	3	4	135,691 (50.0)
	High	5	8	28,987 (10.7)
Combined	Low	− 1.25	0.51	54,292 (20.0)
	Intermediate	0.51	1.37	162,875 (60.0)
	High	1.37	4.33	54,292 (20.0)

## Discussion

In this study, we quantified the risk of venous thromboembolism by examining both clinical and genetic risk factors in U.K. Biobank, a large prospective cohort of over 500,000 participants. We found several noteworthy associations, and we subsequently derived an accurate, novel risk score that combines both clinical and genetic factors. These findings are applicable in a general population setting and support four conclusions.

First, in analyses of clinical risk factors, we found that participants who had ever used oral contraceptives were at decreased risk of venous thromboembolism (HR, 0.88). This finding is in direct contrast to the well-described increased risk of venous thromboembolism with oral contraceptive use^39^. The reasons for this finding are likely multifold. Importantly, while the majority of women had reported ever using oral contraceptive pills, most women had discontinued use by the start of the study. Furthermore, the mean age of participants in our cohort at baseline was over 56 years, suggesting that women who had ever used oral contraceptive pills were decades past the high-risk period for venous thromboembolism that occurs during the first months of oral contraceptive use. Finally, due to contraindications, participants with severe thrombophilias likely did not use oral contraceptive pills.

Second, venous thromboembolism prediction accuracy is significantly improved by considering both the clinical and genetic score over the clinical score alone. Even after participants are categorized according to their clinical risk, according to six major risk factors for venous thromboembolism, a polygenic risk score allows for further categorization of participants into significantly different risk groups. These data indicate that genetic risk factors are powerful modulators of susceptibility to venous thromboembolism, and our results suggest that adoption of polygenic risk scores in the clinic may improve venous thromboembolism prediction and prophylaxis. However, this conclusion is currently only applicable to populations of European ancestry. Further validation is required before the score can be applied to broader populations.

Third, combining clinical and genetic factors into a single combined score yielded a predictive model with diagnostic accuracy (concordance, 0.69) superior to other models. In 2012, *De Haan *et al*.* proposed the use of a combined model of genetic and nongenetic factors to predict primary deep vein thromboses, with or without pulmonary embolism^38^. The group’s nongenetic score included nine factors: leg injury, surgery, pregnancy, immobilization, extended travel, oral contraceptive use, hormone replacement therapy, obesity, and recent cancer diagnosis. De Haan and colleagues found that their 31 SNP score led to an AUC of 0.64, while their nongenetic score led to an AUC of 0.77. The application of the genetic score proposed by *De Haan *et al*.* in this study led to an AUC of 0.54. In order to improve on the study by *De Haan *et al*.*, we used a larger sample size, a longitudinal cohort design accounting for competing events, and the most recent data from large genome wide association studies. Beyond *de Haan *et al*.*’s model, other models exist, although many are only applicable to specific clinical settings.

Fourth, the clinical, genetic, and combined scores are continuous measures of risk that may be used to guide decisions regarding targeted prophylaxis. For example, different levels of risk could indicate the use of different therapies, from no prophylaxis to therapeutic heparin. The pooled cohort equation for cardiovascular disease indicates that moderate- or high-intensity statin therapy is indicated when atherosclerotic cardiovascular disease risk is at least 7.5% over a 10-year period^40^. Similarly, in patients with at least a 4% risk of venous thromboembolism over a 10-year period, targeted prophylaxis may be beneficial. While many prophylactic interventions for venous thromboembolism are known to increase bleeding risk, some interventions like exercise and statin use have been shown to decrease risk of venous thromboembolism without significant increases in risk of bleeds^41,42^. Because the combined score in this study can be easily computed at any time, individualized prophylaxis can be recommended before high risk events such as extended immobility or major surgery. The relatively complex combined score has drawbacks including its price and the time to results, which is generally several weeks for genetic testing. It is possible that resources used for obtaining the score could be attenuated by the diminution of patients requiring prophylaxis. Further reductions in cost could be achieved by using a simple clinical assessment of the patient, using the clinical risk score alone.

### Limitations

Our study has several limitations. First, when using a causal modeling approach on the full dataset, although we attempted to control for confounding through multivariable modeling, residual confounding remains, from variables such as immobility and diet. Second, this study relied on a single cohort of primarily white participants from a single country, which may have resulted in predictive models that overestimate the true power of our scoring system. Third, analyses of the association of medication use with incident venous thromboembolism events were likely subject to indication bias.

## Conclusions

In conclusion, analysis of thromboembolic events in over 500,000 participants identified several known and novel associations. Furthermore, combining genetic and clinical risk factors into a single combined score identified that participants in the top two deciles of the score were at nearly eightfold increased risk of venous thromboembolism relative to participants in the lowest two deciles.

## Supplementary Information

Supplementary Information.

## Data Availability

Data from the UK Biobank is available upon application and is open to any researcher upon request. Data is available from the UK Biobank upon approval.
