# Publisher Correction: Prediction of primary venous thromboembolism based on clinical and genetic factors within the U.K. Biobank

**DOI:** 10.1038/s41598-021-02115-3

**Published:** 2021-11-29

**Authors:** David A. Kolin, Scott Kulm, Olivier Elemento

**Affiliations:** 1 Caryl and Israel Englander Institute for Precision Medicine, Weill Cornell Medicine, New York, NY USA; 2 Physiology, Biophysics, and Systems Biology, Weill Cornell Medicine, New York, NY USA

Correction to: *Scientific Reports* https://doi.org/10.1038/s41598-021-00796-4, published online 01 November 2021

The original version of this Article contained an error in Figure 2, where the dashed vertical line located at the label ‘Adjusted Hazard Ratio for Venous Thromboembolism Event’ inadvertently continued and covered the x-axis label ‘1’ in panel (A) and panel (B).

Furthermore, the order of the Figures was incorrect. Figures 3 and 4 were published as Figure 4 and 3. The Figure legends were correct at the time of publication.

The original Figure 2, 3 and 4 and accompanying legends appear below.

**Figure 2:**
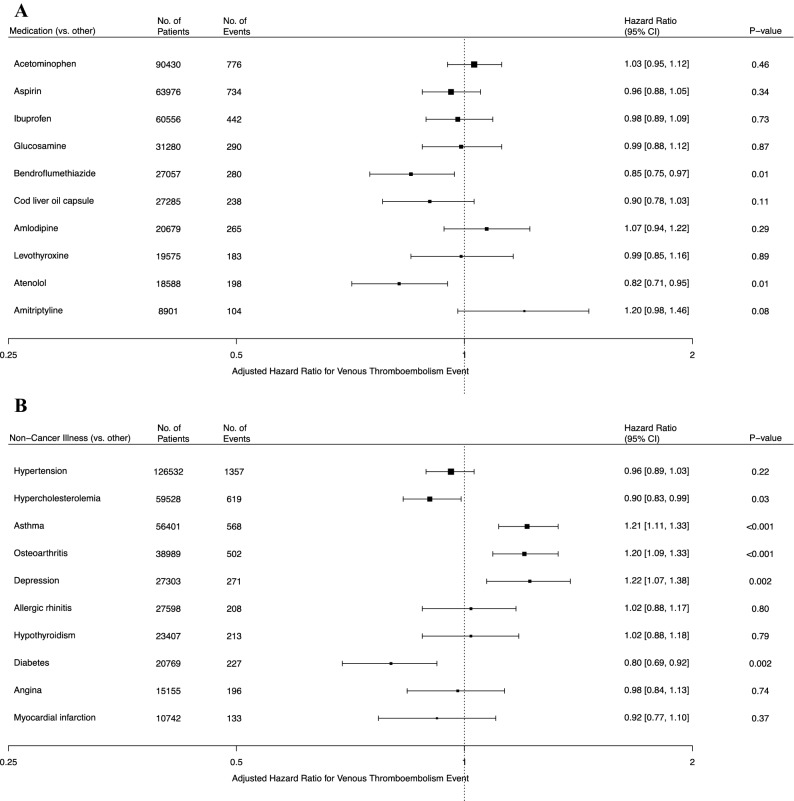
Adjusted hazard ratios for primary venous thromboembolism for common medications and non-cancer illnesses. Shown are the adjusted hazard ratios for primary venous thromboembolic events for two factors of interest: common medications (**A**) and non-cancer illnesses (**B**). All estimates were adjusted for all nine established risk factors and the first four principal components of ancestry. The I bars represent 95% confidence intervals.

**Figure 3:**
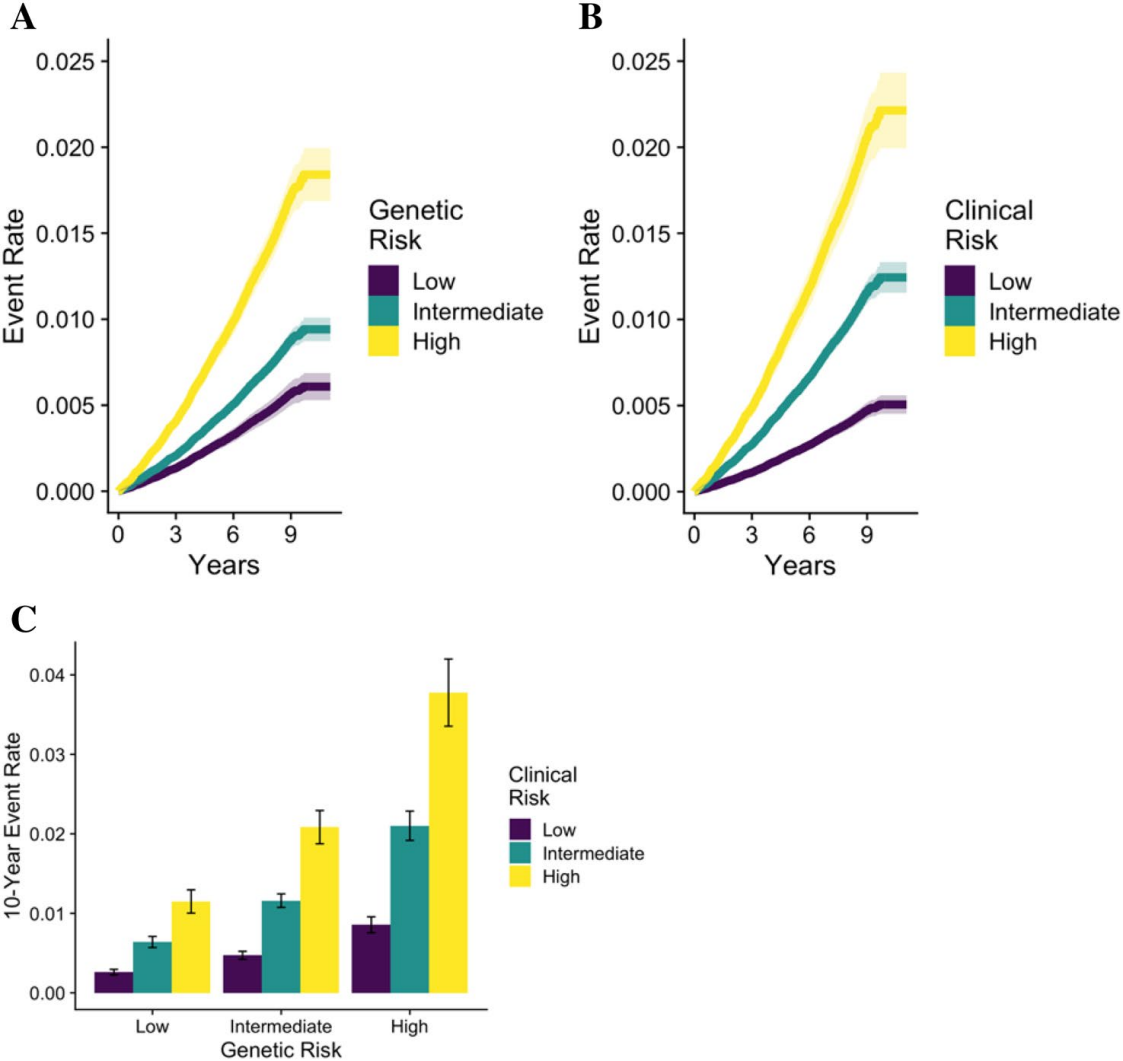
Model performance of a genetic, clinical, and combined score. (**A**) Shows the density of the polygenic risk score stratified by the 50th, 75th, 90th, 95th, 99th and 99.5th percentiles. (**B**) Shows the odds ratio for each polygenic risk score percentile. (**C**) Shows the concordance values, derived from Fine-Gray models, for the De Haan, genetic, clinical, and combined scores. The concordance of the combined model is significantly higher than the concordance of each of the other models (*P* < 0.001). The I bars represent 95% confidence intervals.

**Figure 4:**
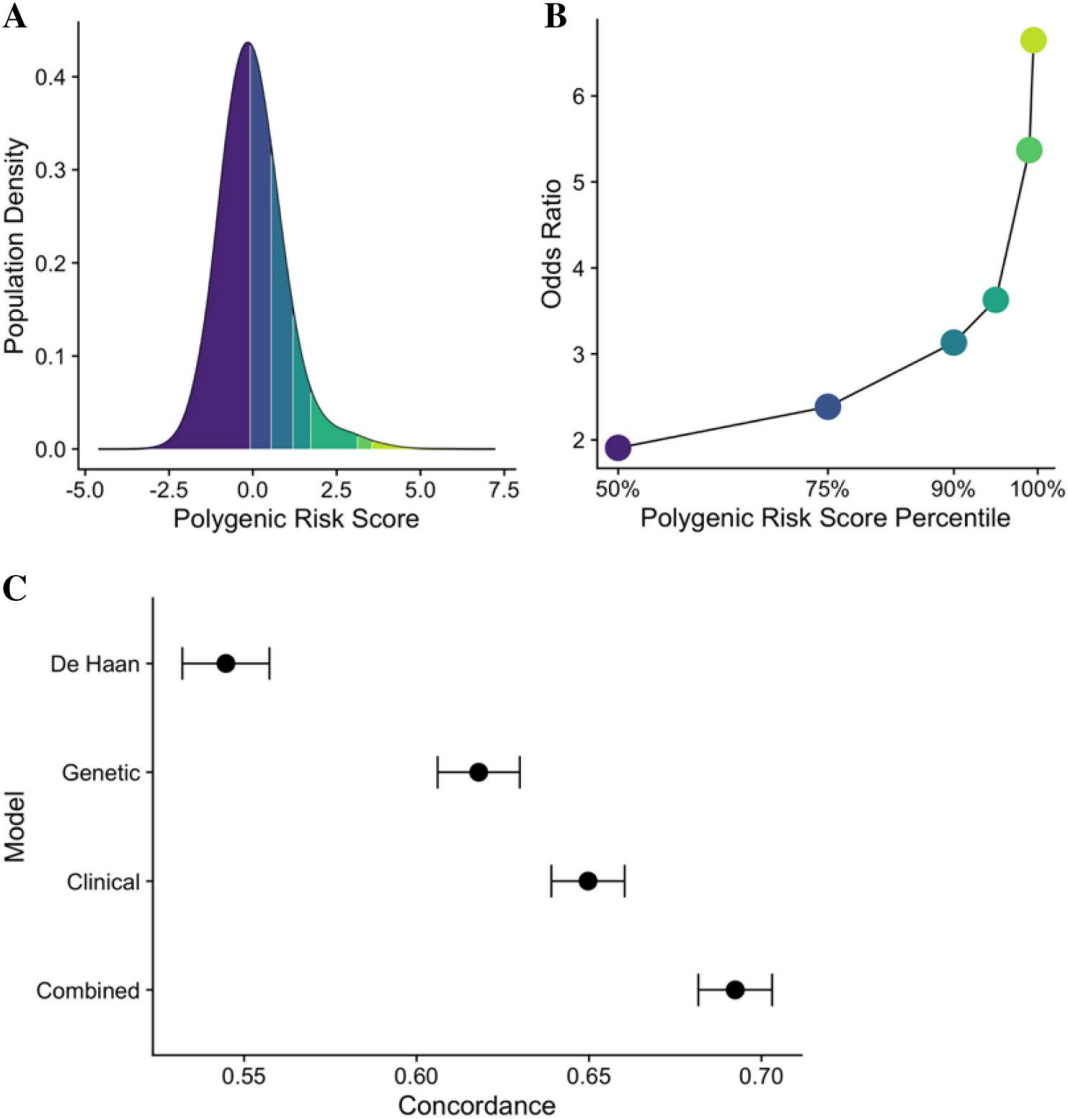
Prediction of venous thromboembolism. (**A**) and (**B**) Show the rates of venous thromboembolism derived from Fine-Gray models for the genetic and clinical score, respectively. (**C**) Shows 10-year event rates for venous thromboembolism, stratified by both the clinical score and genetic score. The I bars represent 95% confidence intervals.

The original Article has been corrected.

