# Supplementary material for: Prediction of primary venous thromboembolism based on clinical and genetic factors within the U.K. Biobank
Source: Sci Rep. 2021 Nov 1;11:21340. doi: 10.1038/s41598-021-00796-4 (PMC8560817; doi:10.1038/s41598-021-00796-4)
Supplement: Supplementary file 1 — Supplementary Information. [file 41598_2021_796_MOESM1_ESM.docx]

**Supplement 1**

**Prediction of Primary Venous Thromboembolism Based on Clinical and Genetic Factors within the U.K. Biobank**

David A. Kolin, M.Sc.*; Scott Kulm, B.Eng.*; Olivier Elemento, Ph.D.

*Mr. Kolin and Kulm contributed equally to this article.

Address for Correspondence:

Olivier Elemento, Ph.D.

Caryl and Israel Englander Institute for Precision Medicine

The Meyer Cancer Center

Weill Cornell Medicine

1300 York Avenue

New York, NY 10065

ole2001@med.cornell.edu

**Table of Contents:**

**Page 3 eTable 1.** Definition of Venous Thromboembolism

**Page 4 eTable 2.** Single Nucleotide Polymorphisms Used to Calculate the Polygenic Risk Score

**Page 5 eTable 3.** Clinical Risk Score for Venous Thromboembolism Based on Established Risk Factors

**Page 6 eTable 4.** R Packages Utilized

**Page 7 eTable 5.** Characteristics of the Participants at Baseline

**Page 8 eTable 6.** Risk of Venous Thromboembolism with Duration of Contraception Use

**Page 9 eTable 7.** The Five Most Common Oral Contraceptive Pills Amongst Current Users

**Page 10 eTable 8.** Univariate Risk Ratios for Death from Venous Thromboembolism

**Page 11 eTable 9.** Risk of Venous Thromboembolism with Common Non-Cancer Illnesses, Adjusted for Common Medications

**Page 12 eTable 10.** Comparison of the Predictors Within the Training and Testing Phases

**Page 13 eTable 11.** Count of participants with Missing Predictors

**Page 14 eTable 12.** Coefficients Comprising the Full Prediction Models

**Page 15 eTable 13.** Coefficients of Cubic Functions that Approximate the Baseline Hazard Function

**Page 16 eFigure 1.** Cumulative Incidence Curves for Four Individual Clinical Factors

**Page 17 eFigure 2.** Fully Adjusted Risk of Venous Thromboembolism with Common Cancer Subtypes

**Page 18 eFigure 3.** Fully Adjusted Risk of Venous Thromboembolism with Common Fracture Sites

**Page 19 eFigure 4.** Fully Adjusted Hazard Ratio of Any Venous Thromboembolism with Established Clinical Risk Factors

**Page 20 eFigure 5.** Fully Adjusted Hazard Ratio of Any Venous Thromboembolism with Common Medications

**Page 21 eFigure 6.** Fully Adjusted Hazard Ratio of Any Venous Thromboembolism with Common Non-Cancer Illnesses

**Page 22 eFigure 7.** Fully Adjusted Hazard Ratio of Any Venous Thromboembolism with Common Cancer Subtypes

**Page 23 eFigure 8.** Fully Adjusted Hazard Ratio of Any Venous Thromboembolism with Common Fracture Sites

**Page 24 eFigure 9.** Minimally Adjusted Hazard Ratio of Any Venous Thromboembolism with Established Clinical Risk Factors

**Page 25 eFigure 10.** Minimally Adjusted Hazard Ratio of Any Venous Thromboembolism with Common Cancer Subtypes

**Page 26 eFigure 11.** Minimally Adjusted Hazard Ratio of Any Venous Thromboembolism with Common Medications

**Page 27 eFigure 12.** Minimally Adjusted Hazard Ratio of Any Venous Thromboembolism with Common Non-Cancer Illnesses

**Page 28 eFigure 13.** Minimally Adjusted Hazard Ratio of Any Venous Thromboembolism with Common Fracture Sites

**Page 29 eFigure 14.** Stratification of the polygenic risk score by age and sex

**Page 30 eFigure 15.** Subhazard Ratios for Sub-Classified Venous Thromboembolic Events with the Clinical and Genetic Scores

**Page 31 eFigure 16.** Interactions Between Individual Covariates and the Genetic Score

**Page 32** **eFigure 17.** Associations Derived from a Cox-proportional Hazard Model for Possible Frailty within the Population

**Page 33 eFigure 18.** Schoenfeld Residuals for Venous Thromboembolism at Various Times

**Page 34 eFigure 19.** Analysis of Fine-Gray Proportional Hazards Assumption

**Page 35 eFigure 20.** Comparison of Fine-Gray and Cox Proportional Hazard Model Results

**Page 36 eFigure 21.** Cumulative Incidence Curve of the Combined Score

**eTable 1.** Definition of Venous Thromboembolism

| **Outcome** | **ICD-10** | **ICD-9** |
| --- | --- | --- |
| **Venous thromboembolism** |  |  |
| **Pulmonary embolism (PE)** | I26 | 415 |
| **Deep vein thrombosis (DVT)** | I80-I82 | 451-453 |

**eTable 2.** Single Nucleotide Polymorphisms Used to Calculate the Polygenic Risk Score

| **Chromosome** | **Position** | **P-value** | **Effect** | **Allele** | **rs ID** | **Study** |
| --- | --- | --- | --- | --- | --- | --- |
| 1 | 169542517 | 3.00 x 10^-11^ | 0.066 | T | rs4524 | Germain, 2015 |
| 1 | 169472899 | 6.99 x 10^-9^ | -0.008 | G | rs6670848 | De Haan, 2018 |
| 1 | 169481121 | 7.92 x 10^-9^ | -0.012 | A | rs6427194 | De Haan, 2018 |
| 1 | 159902335 | 5.07 x 10^-6^ | 0.058 | G | rs111438240 | De Haan, 2018 |
| 1 | 169435027 | 1.43 x 10^-7^ | 0.516 | A | rs2678166 | De Haan, 2018 |
| 1 | 169549811 | 1.00 x  10^-300^ | 0.012 | A | rs6025 | Klarin, 2019 |
| 1 | 169544768 | 2.00 x 10^-26^ | 0.189 | T | rs1018827 | Germain, 2011 |
| 2 | 42372465 | 2.00 x 10^-7^ | -0.077 | G | rs72798544 | Hinds, 2016 |
| 2 | 164295170 | 4.14 x 10^-6^ | -0.050 | A | rs144482539 | De Haan, 2018 |
| 3 | 10571102 | 2.48 x 10^-6^ | 0.047 | A | rs34029315 | De Haan, 2018 |
| 4 | 154599778 | 2.00 x 10^-13^ | 0.043 | A | rs7659024 | Germain, 2011 |
| 4 | 154622217 | 2.00 x 10^-13^ | 0.026 | C | rs6536024 | Wang, 2013 |
| 4 | 154604124 | 2.00 x 10^-88^ | 0.074 | A | rs2066865 | Klarin, 2019 |
| 4 | 186277851 | 8.00 x 10^-96^ | 0.101 | C | rs4253417 | Klarin, 2019 |
| 5 | 135637432 | 2.07 x 10^-6^ | 0.063 | G | rs142454359 | De Haan, 2018 |
| 5 | 38708452 | 3.00 x 10^-10^ | 0.006 | C | rs16867574 | Klarin, 2019 |
| 6 | 8859837 | 6.01 x 10^-6^ | -0.0004 | C | rs78069640 | De Haan, 2018 |
| 8 | 105578478 | 5.00 x 10^-7^ | 0.045 | A | rs4602861 | Germain, 2015 |
| 8 | 105570896 | 4.00 x 10^-19^ | 0.065 | A | rs4734879 | Klarin, 2019 |
| 9 | 133279294 | 3.00 x 10^-16^ | 0.118 | T | rs495828 | Heit, 2012 |
| 9 | 112060841 | 3.00 x 10^-6^ | -0.021 | C | rs4979078 | Wang, 2013 |
| 9 | 39158211 | 9.29 x 10^-6^ | 0.011 | A | rs115361037 | De Haan, 2018 |
| 9 | 133269992 | 1.00 x 10^-224^ | -0.101 | A | rs9411377 | Klarin, 2019 |
| 10 | 69458890 | 3.00 x 10^-7^ | 0.201 | G | rs17490626 | Hinds, 2016 |
| 10 | 119250744 | 2.00 x 10^-18^ | 0.072 | G | rs10886430 | Klarin, 2019 |
| 11 | 46739505 | 1.00 x 10^-59^ | 0.233 | A | rs1799963 | Klarin, 2019 |
| 12 | 113076475 | 8.75 x 10^-6^ | -0.038 | C | rs4766986 | De Haan, 2018 |
| 14 | 91768695 | 9.00 x 10^-10^ | 0.017 | G | rs57328376 | Klarin, 2019 |
| 15 | 31501727 | 3.00 x 10^-6^ | -0.018 | A | rs7164569 | Wang, 2013 |
| 16 | 81837364 | 2.00 x 10^-21^ | 0.048 | T | rs12445050 | Klarin, 2019 |
| 18 | 65804614 | 6.91 x 10^-6^ | 0.037 | G | rs150366483 | De Haan, 2018 |
| 19 | 10632450 | 6.00 x 10^-9^ | 0.076 | T | rs9797861 | Hinds, 2016 |
| 19 | 816919 | 3.80 x 10^-6^ | -0.003 | C | rs61504683 | De Haan, 2018 |
| 20 | 1191140 | 5.86 x 10^-6^ | -0.004 | T | rs126622 | De Haan, 2018 |
| 20 | 23201922 | 7.00 x 10^-12^ | 0.026 | A | rs6083037 | Klarin, 2019 |
| 20 | 35187566 | 4.00 x 10^-32^ | 0.044 | A | rs10747514 | Klarin, 2019 |

**eTable 3.** Clinical Risk Score for Venous Thromboembolism Based on Established Risk Factors

| **Clinical Risk Factors** | **Points (Total = 8)** |
| --- | --- |
| **Sex** | Female – 0; Male – 1 |
| **Age, years** | <50 – 0; ≥50 to <60 – 1; ≥60 – 2 |
| **Body mass index, kg/m^2^** | <25 – 0; ≥25 to <30 – 1; ≥30 – 2 |
| **Cancer** | No – 0; Yes – 1 |
| **Smoking** | Not current – 0; Current – 1 |
| **Fracture in the last five years** | No – 0; Yes – 1 |

**eTable 4.** R Packages Utilized

| **Package Name** | **Version** |
| --- | --- |
| stringr | 1.4.0 |
| viridis | 0.5.1 |
| gridExtra | 2.3 |
| reshape2 | 1.4.3 |
| cowplot | 1.0.0 |
| ggplot2 | 3.2.1 |
| risksetROC | 1.0.4 |
| MASS | 7.3-49 |
| timereg | 1.9.4 |
| pROC | 4.2-0 |
| rms | 5.1-3.1 |
| pec | 2018.07.26 |
| survival | 2.41-3 |
| survminer | 0.4.6 |

These are the primary R packages called in the statistical analysis. Additional packages were also utilized as dependencies of those listed.

**eTable 5.** Characteristics of the Participants at Baseline

| **Variable** | **Total (N = 502,536)** |
| --- | --- |
| **Sex ­– no. (%)** |  |
| **Female** | 273,402 (54.4) |
| **Male** | 229,134 (45.6) |
| **Age, years – mean (95% CI)** | 56.53 (56.51-56.55) |
| **Body mass index, kg/m^2^ – mean (95% CI)** | 27.43 (27.42-27.45) |
| **Previous cancer diagnosis – no. (%)** |  |
| **No** | 459,974 (91.5) |
| **Yes** | 41,700 (8.3) |
| **Unknown** | 862 (0.2) |
| **Smoking status – no. (%)** |  |
| **Never** | 273,537 (54.4) |
| **Previous** | 173,030 (34.4) |
| **Current** | 52,979 (10.5) |
| **Declined to answer** | 2059 (0.4) |
| **Unknown** | 891 (0.2) |
| **Alcohol intake frequency – no. (%)** |  |
| **Never** | 40,648 (8.1) |
| **Special occasions only** | 58,012 (11.5) |
| **One to three times a month** | 55,858 (11.1) |
| **Once or twice a week** | 129,297 (25.7) |
| **Three or four times a week** | 115,445 (23.0) |
| **Daily or almost daily** | 101,774 (20.3) |
| **Prefer not to answer** | 605 (0.1) |
| **Unknown** | 897 (0.2) |
| **Ever use oral contraceptive pill – no. (%)** |  |
| **No** | 51,532 (18.8) |
| **Yes** | 220,446 (80.6) |
| **Do not know** | 444 (0.2) |
| **Prefer not to answer** | 497 (0.2) |
| **Unknown** | 483 (0.2) |
| **Ever use hormone replacement therapy – no. (%)** |  |
| **No** | 167,903 (61.4) |
| **Yes** | 103,919 (38.0) |
| **Do not know** | 800 (0.3) |
| **Prefer not to answer** | 297 (0.1) |
| **Unknown** | 483 (0.2) |
| **Fracture in the last 5 years – no. (%)** |  |
| **No** | 451,266 (90.0) |
| **Yes** | 47,466 (9.4) |
| **Do not know** | 2,533 (0.5) |
| **Declined to answer** | 340 (<0.1) |
| **Unknown** | 931 (0.2) |

**eTable 6.** Risk of Venous Thromboembolism with Duration of Contraception Use

| **Duration of Contraception Use (Years)** | **No. of Patients** | **No. of Events** | **Hazard Ratio (95% CI)** | **P-value** |
| --- | --- | --- | --- | --- |
| **Other** | 77,900 | 820 | 1 | 0.24 |
| **>0 to <10** | 99,601 | 779 | 0.94 (0.84-1.05) |  |
| **≥10 to <20** | 66,377 | 408 | 0.88 (0.77-1.01) |  |
| **≥20** | 29,524 | 151 | 0.80 (0.65-0.98) |  |

Cox regression models were adjusted for sex, age, body mass index, previous cancer diagnosis, smoking status, alcohol intake frequency, use of hormone replacement therapy, fracture in the last five years, previous deep vein thrombosis, previous pulmonary embolism, and the first four principal components of ancestry. The p-value was calculated with duration of contraception use coded as a continuous variable.

**eTable 7.** The Five Most Common Oral Contraceptive Pills Amongst Current Users

| **Oral Contraceptive Pill** | **Total (N = 2,598)** |
| --- | --- |
| **Cerazette (75 μg tablet)** | 935 |
| **Micronor tablet** | 558 |
| **Microgynon 30 tablet** | 518 |
| **Femulen tablet** | 305 |
| **Noriday tablet** | 282 |

**eTable 8.** Univariate Risk Ratios for Death from Venous Thromboembolism

| **Variable** | **No. of Patients** | **No. of Events** | **Risk Ratio (95% CI)** |
| --- | --- | --- | --- |
| **Sex** |  |  |  |
| **Female** | 273,402 | 102 | 1 |
| **Male** | 229,134 | 133 | 1.56 (1.20-2.01) |
| **Age, years** |  |  |  |
| **<50** | 117,885 | 21 | 1 |
| **≥50 to <60** | 167,158 | 60 | 2.01 (1.23-3.31) |
| **≥60** | 217,493 | 154 | 3.97 (2.52-6.27) |
| **Body-mass index, kg/m^2^** |  |  |  |
| **<18.5** | 2,626 | 3 | 5.46 (1.68-17.76) |
| **≥18.5 to <25** | 162,423 | 34 | 1 |
| **≥25 to <30** | 212,127 | 79 | 1.78 (1.19-2.66) |
| **≥30 to <35** | 87,557 | 60 | 3.27 (2.15-4.99) |
| **≥35 to <40** | 24,994 | 26 | 4.97 (2.98-8.28) |
| **≥40** | 9,704 | 28 | 13.78 (8.36-22.72) |
| **Previous cancer diagnosis** |  |  |  |
| **No** | 459,974 | 211 | 1 |
| **Yes** | 41,700 | 23 | 1.20 (0.78-1.85) |
| **Ever smoked** |  |  |  |
| **Never** | 273,537 | 113 | 1 |
| **Previous** | 173,070 | 80 | 1.12 (0.84-1.49) |
| **Current** | 52,979 | 38 | 1.74 (1.20-2.51) |
| **Alcohol intake frequency** |  |  |  |
| **Never** | 40,648 | 26 | 1 |
| **Special occasions only** | 58,012 | 47 | 1.27 (0.78-2.04) |
| **One to three times a month** | 55,858 | 28 | 0.78 (0.46-1.34) |
| **Once or twice a week** | 129,297 | 53 | 0.64 (0.40-1.02) |
| **Three or four times a week** | 115,445 | 43 | 0.58 (0.36-0.95) |
| **Daily or almost daily** | 101,774 | 37 | 0.57 (0.34-0.94) |
| **Ever use oral contraceptive pill** |  |  |  |
| **No** | 51,532 | 31 | 1 |
| **Yes** | 220,446 | 69 | 0.52 (0.34-0.79) |
| **Ever use hormone-replacement therapy** |  |  |  |
| **No** | 167,903 | 52 | 1 |
| **Yes** | 103,919 | 49 | 1.52 (1.03-2.25) |
| **Fracture in the last 5 years** |  |  |  |
| **No** | 451,266 | 214 | 1 |
| **Yes** | 47,466 | 20 | 0.89 (0.56-1.40) |
| **Previous deep veins thrombosis** |  |  |  |
| **No** | 492,582 | 219 | 1 |
| **Yes** | 9,323 | 16 | 3.86 (2.32-6.41) |
| **Previous pulmonary embolism** |  |  |  |
| **No** | 498,417 | 221 | 1 |
| **Yes** | 3,955 | 14 | 7.98 (4.66-13.69) |

**eTable 9.** Risk of Venous Thromboembolism with Common Non-Cancer Illnesses, Adjusted for Common Medications

| **Common Non-Cancer Illness** | **Additional Covariates** | **Hazard Ratio**  **(95% CI)** |
| --- | --- | --- |
| **Hypercholesterolemia** | Atorvastatin, simvastatin, and pravastatin | 0.99 (0.88-1.11) |
| **Asthma** | Beclomethasone, beconase, and becotide | 1.19 (1.08-1.31) |
| **Osteoarthritis** | Aspirin, ibuprofen, and diclofenac | 1.19 (1.08-1.32) |
| **Depression** | Amitriptyline, citalopram, and fluoxetine | 1.21 (1.04-1.40) |
| **Diabetes** | Metformin, insulin products, and glibenclamide | 0.87 (0.72-1.05) |

Cox regression models were adjusted for sex, age, body mass index, previous cancer diagnosis, smoking status, alcohol intake frequency, use of oral contraceptive pills, use of hormone replacement therapy, fracture in the last five years, previous deep vein thrombosis, previous pulmonary embolism, and the first four principal components of ancestry. Common illness-specific medications were added to each model to test mediation by medication use.

**eTable 10.** Comparison of the Predictors Within the Training and Testing Phases

| **Predictor** | **Train** | **Test** | **P-Value** |
| --- | --- | --- | --- |
| Age* | 56.8 | 56.8 | 0.725 |
| Sex (Male) | 0.473 | 0.455 | 1.06e-26 |
| BMI* | 27.5 | 27.3 | 1.51e-29 |
| Cancer | 0.916 | 0.916 | 0.99 |
| Alcohol Consumption - Daily | 0.213 | 0.212 | 0.364 |
| Alcohol Consumption - Weekly | 0.263 | 0.266 | 0.028 |
| Alcohol Consumption - Never | 0.0673 | 0.0633 | 2.68e-06 |
| Smoking Status - Never | 0.536 | 0.552 | 4e-23 |
| Smoking Status - Previous | 0.341 | 0.357 | 4.96e-23 |
| Smoking Status - Current | 0.123 | 0.0903 | 1.23e-218 |
| Fractures | 0.0958 | 0.0942 | 0.103 |
| Contraceptive Use | 0.827 | 0.825 | 0.174 |
| Hormone Replacement Therapy | 0.399 | 0.393 | 0.00487 |
| PRS* | -0.00961 | -0.0102 | 0.577 |
| Venous Thromboembolism Event | 0.00896 | 0.00845 | 0.113 |

Statistics for each predictor were computed in both the train and testing phases. The statistic for predictors notated with an asterisk is the mean, the statistic for predictors without an asterisk is the proportion of events or individuals reporting the predictor divided by the total number of participants in the phase. These statistics were compared either by a Wilcoxon Rank Sum Test, for the predictors with an asterisk, or Fishers Exact Test, for the predictors without an asterisk. The P-values from these tests are reported.

**eTable 11.** Count of participants with Missing Predictors

| **Predictor** | **Train** | **Test** |
| --- | --- | --- |
| BMI | 867 | 322 |
| Cancer | 34 | 10 |
| Smoker | 1049 | 327 |
| Alcohol | 193 | 82 |
| Fracture | 1261 | 624 |

The number of participants in each phase with a missing feature were enumerated.

**eTable 12.** Coefficients Comprising the Full Prediction Models

| **Model** | **Predictors** | **Coefficient** | **SE** | **P - Value** |
| --- | --- | --- | --- | --- |
| 1 | Clinical Score | 0.36935 | 0.02291 | < 1x10^-16^ |
|  | Genetic Score | 0.38863 | 2.52E-02 | < 1x10^-16^ |
| 2 | Clinical Score | 0.40649 | 1.61E-02 | < 1x10^-16^ |
| 3 | Genetic Score | 0.39118 | 0.01772 | < 1x10^-16^ |
| 4 | Combined Score | 1.06266 | 0.04143 | < 1x10^-16^ |

The coefficients that comprise Fine-Gray models, which were the primary prediction models. The form of the Fine-Gray model is h(t) = h_o_(t) exp(β_1_p_1_ + β_2_P_2_). The p’s denote the predictor values for each participant and the betas are the coefficients defined in the table. For models 1, 3 and 4 there is only one beta term, and for model 2 there are two beta terms.

**eTable 13.** Coefficients of Cubic Functions that Approximate the Baseline Hazard Function

| **Model** | **Intercept** | **Degree 1** | **Degree 2** | **Degree 3** | **Adj. R^2^** |
| --- | --- | --- | --- | --- | --- |
| 1 | 1.71 x10 ^-05^ | 4.80 x10 ^-07^ | 1.03x10^-10^ | 6.98 x10 ^-15^ | 0.9923 |
| 2 | 7.87 x10 ^-05^ | 1.70 x10 ^-07^ | 3.45 x10 ^-10^ | -5.01 x10 ^-14^ | 0.9967 |
| 3 | 2.69 x10 ^-04^ | 5.75 x10 ^-07^ | 1.18 x10 ^-09^ | -1.72 x10 ^-13^ | 0.9967 |
| 4 | 7.62 x10 ^-5^ | 1.62 x10 ^-7^ | 3.32 x10 ^-10^ | -4.81 x10 ^-14^ | 0.9966 |

To fully describe the prediction models the baseline hazard function, a non-parametric function, must be defined. Therefore, an approximation of a cubic function is made to the baseline hazard function. Specifically, this cubic function is of the form h_o_(t) = a_0_ + a_1_t + a_2_t^2^ + a_3_t^3^. The intercept is a_0_, the Degree 1 term is a_1_, and so on. To fully implement the prediction model, the time at which the hazard is desired should be input into the cubic function to get the baseline hazard. This baseline hazard is then multiplied by the coefficients listed in eTable 12 multiplied by the predictors, exponentiated. Lastly, to see if the cubic function is relatively good the adjusted R-squared value is computed and listed.

**eFigure 1.** Cumulative Incidence Curves for Four Individual Clinical Factors

**
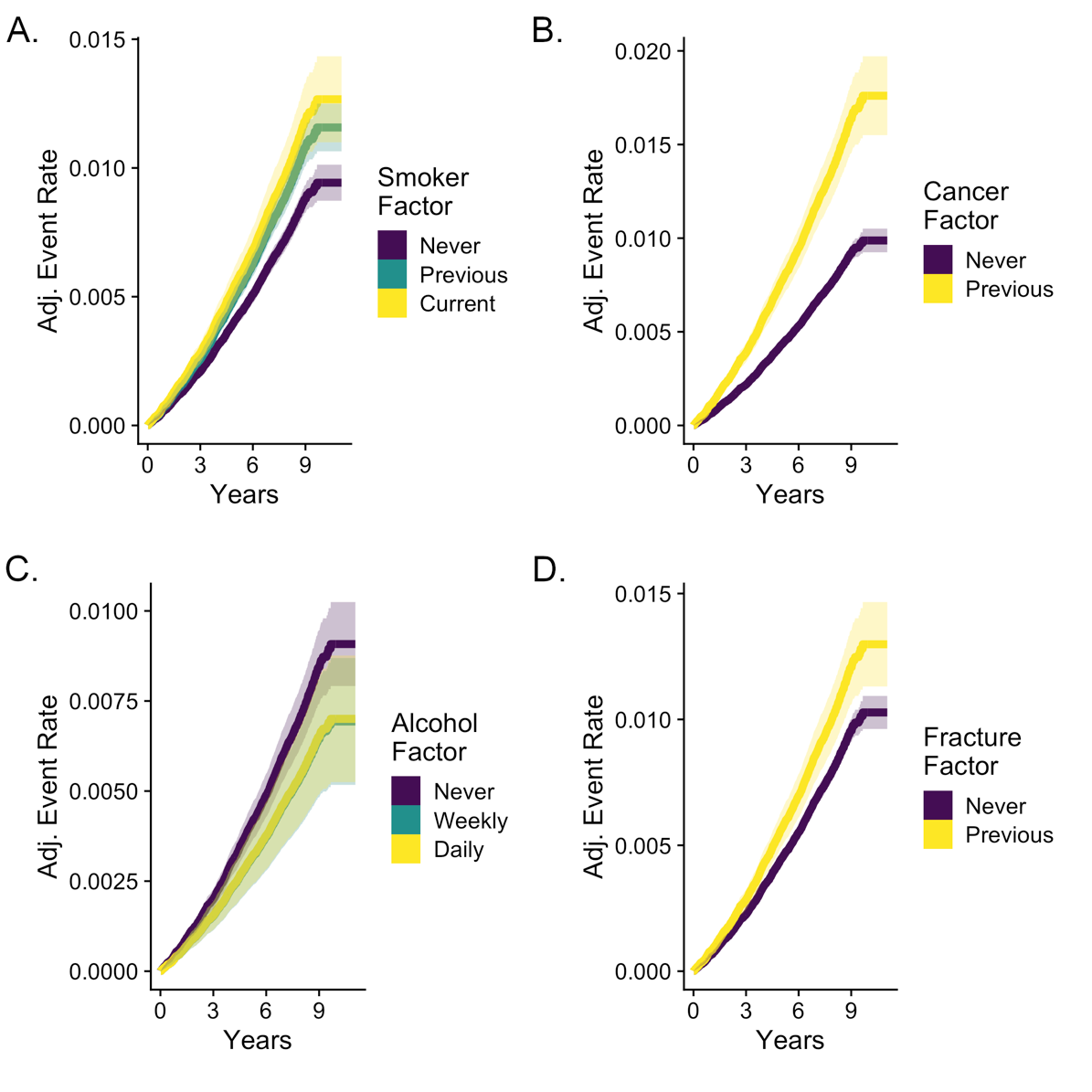
**

The cumulative incidences are generated with the Fine- Gray model adjusted for the other clinical factors that comprised the clinical risk score.

**eFigure 2.** Fully Adjusted Hazard Ratio of Primary Venous Thromboembolism with Common Cancer Subtypes

**
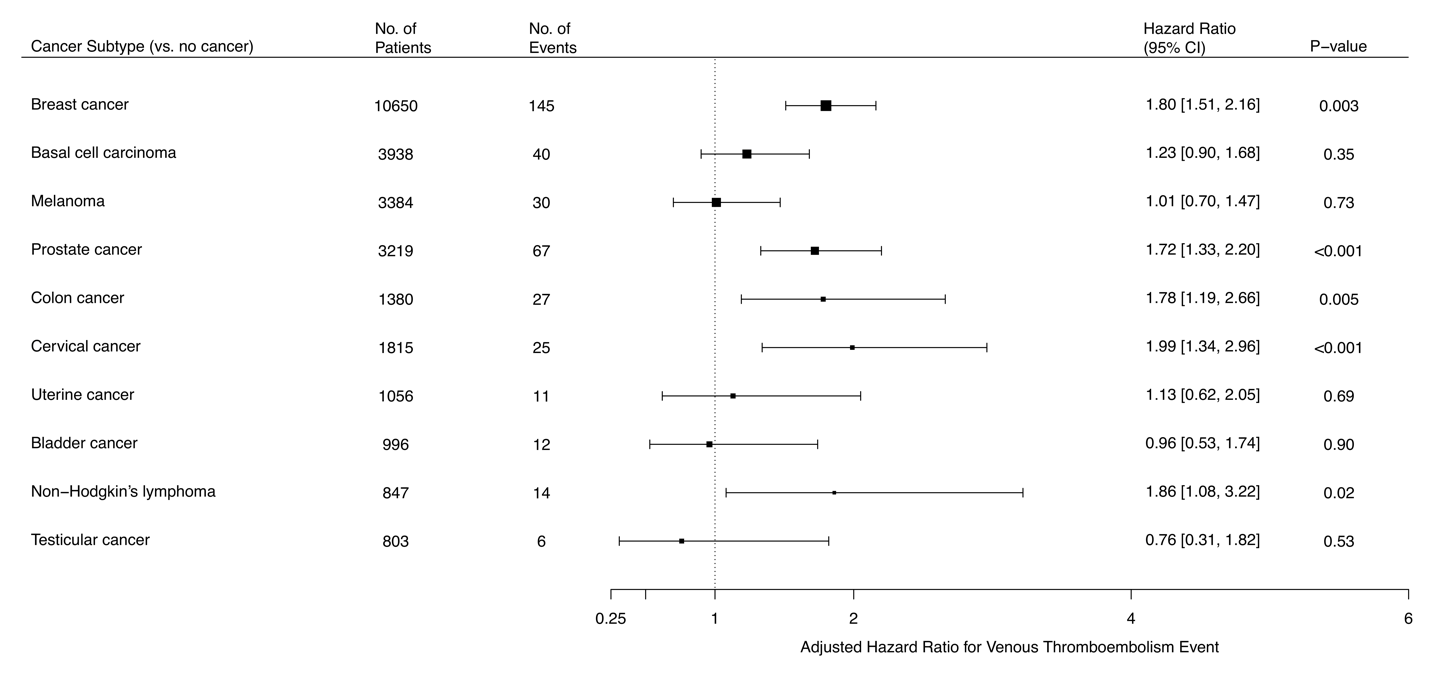
**

**eFigure 3.** Fully Adjusted Hazard Ratio of Primary Venous Thromboembolism with Common Fracture Sites

**
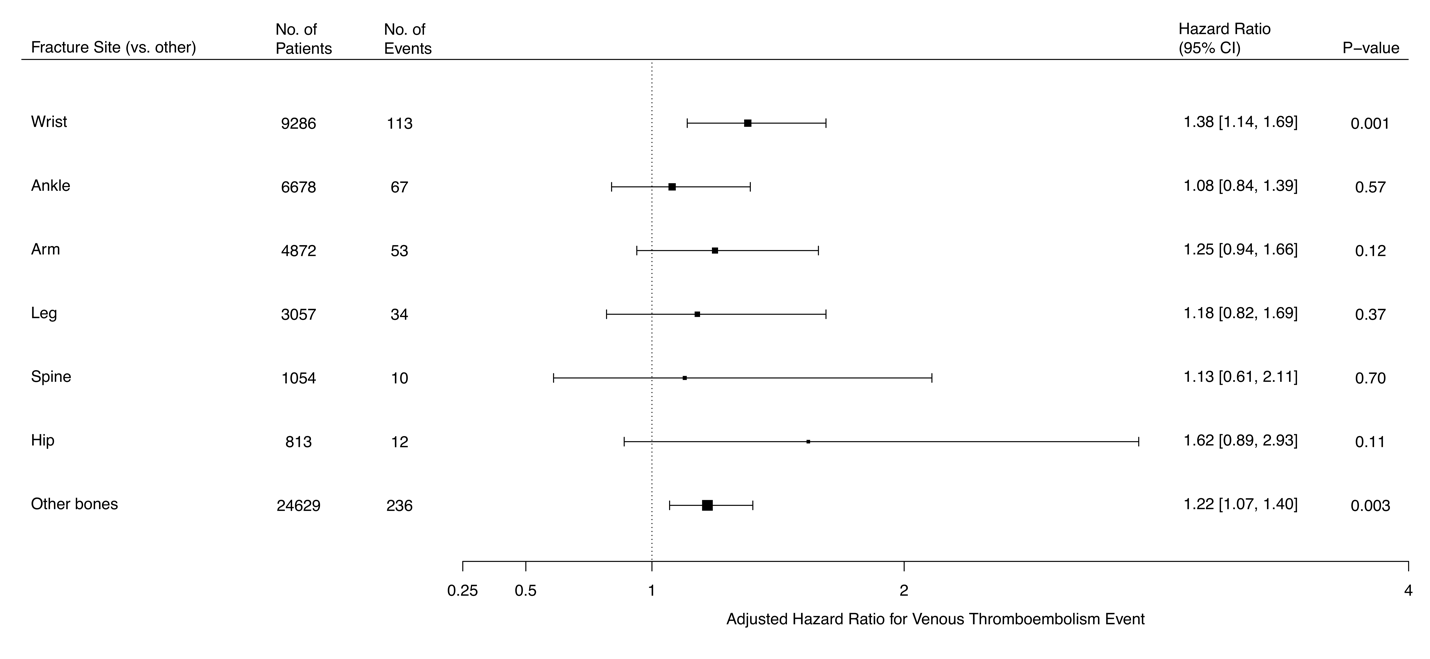
**

**eFigure 4.** Fully Adjusted Hazard Ratio of Any Venous Thromboembolism with Established Clinical Risk Factors

**eFigure 5.** Fully Adjusted Hazard Ratio of Any Venous Thromboembolism with Common Medications

**eFigure 6.** Fully Adjusted Hazard Ratio of Any Venous Thromboembolism with Common Non-Cancer Illnesses

**eFigure 7.** Fully Adjusted Hazard Ratio of Any Venous Thromboembolism with Common Cancer Subtypes

**eFigure 8.** Fully Adjusted Hazard Ratio of Any Venous Thromboembolism with Common Fracture Sites

**eFigure 9.** Minimally Adjusted Hazard Ratio of Any Venous Thromboembolism with Established Clinical Risk Factors

Minimally adjusted Cox regression models were adjusted for sex, age, body mass index, and smoking status.

**eFigure 10.** Minimally Adjusted Hazard Ratio of Any Venous Thromboembolism with Common Cancer Subtypes

Minimally adjusted Cox regression models were adjusted for sex, age, body mass index, and smoking status.

**eFigure 11.** Minimally Adjusted Hazard Ratio of Any Venous Thromboembolism with Common Medications

Minimally adjusted Cox regression models were adjusted for sex, age, body mass index, and smoking status.

**eFigure 12.** Minimally Adjusted Hazard Ratio of Any Venous Thromboembolism with Common Non-Cancer Illnesses

Minimally adjusted Cox regression models were adjusted for sex, age, body mass index, and smoking status.

**eFigure 13.** Minimally Adjusted Hazard Ratio of Any Venous Thromboembolism with Common Fracture Sites

Minimally adjusted Cox regression models were adjusted for sex, age, body mass index, and smoking status.

**eFigure 14.** Stratification of the polygenic risk score by age and sex


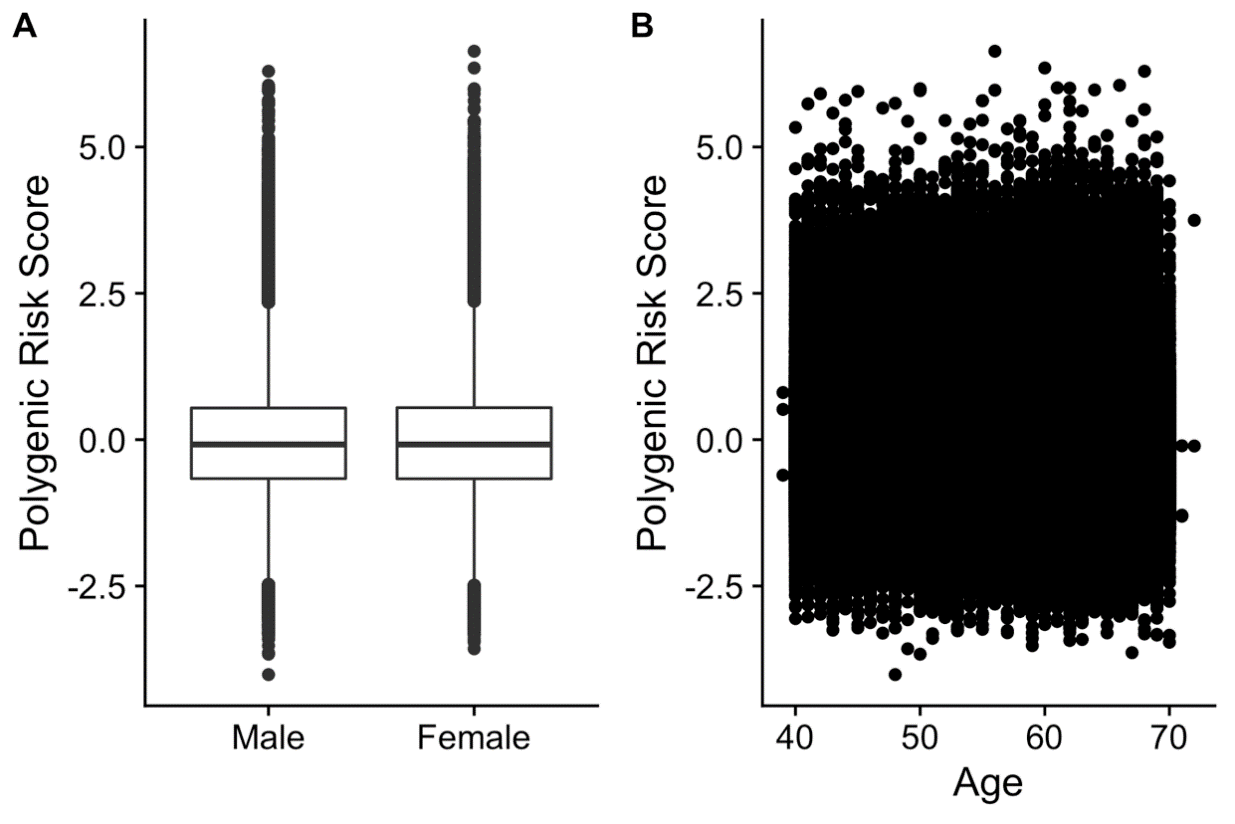


The polygenic risk score is shown to be clearly uncorrelated to both sex and age.

**eFigure 15.** Subhazard Ratios for Sub-Classified Venous Thromboembolic Events with the Clinical and Genetic Scores


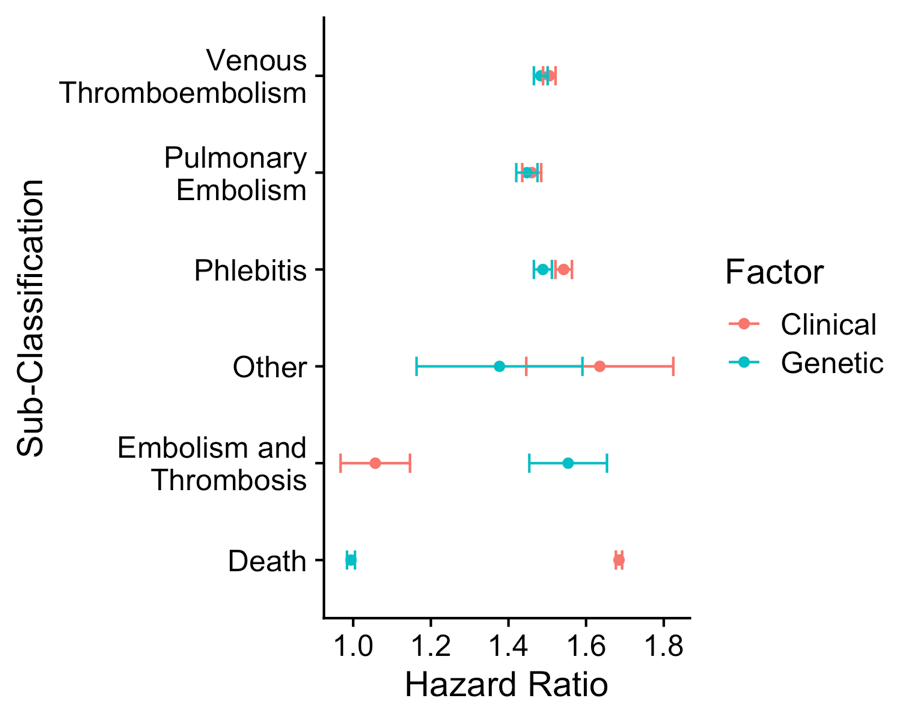


Event sub-classifications were coded as follows: pulmonary embolism (I26), phlebitis (I80), other (I81), and embolism and thrombosis (I82). Subhazard ratios were calculated with the Fine-Gray model, simultaneously evaluating clinical and genetic scores coded on a continuous scale. Error bars denote the standard error.

**eFigure 16.** Interactions Between Individual Covariates and the Genetic Score


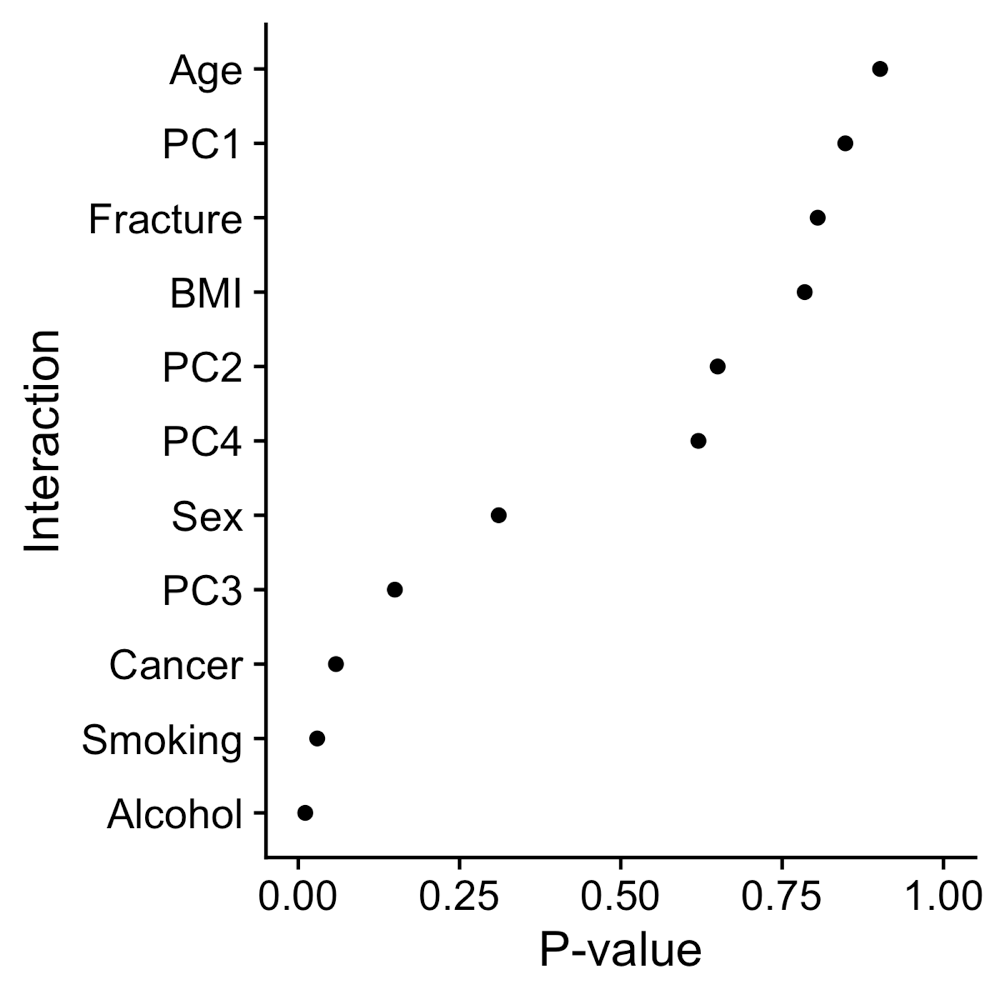


Interactions were tested between the individual covariates indicated and the genetic score. The p-values for interactions were computed through an ANOVA test between a Fine-Gray model of the genetic and clinical score alone and a Fine-Gray model of the genetic and clinical score, along with the interaction term. The smallest interaction p-value is 0.029, above the Bonferroni Correction threshold of 0.0045, thereby indicating there were not any potential, unaccounted for interactions between the model terms and the polygenic risk score.

**eFigure 17.** Associations Derived from a Cox-proportional Hazard Model for Possible Frailty within the Population


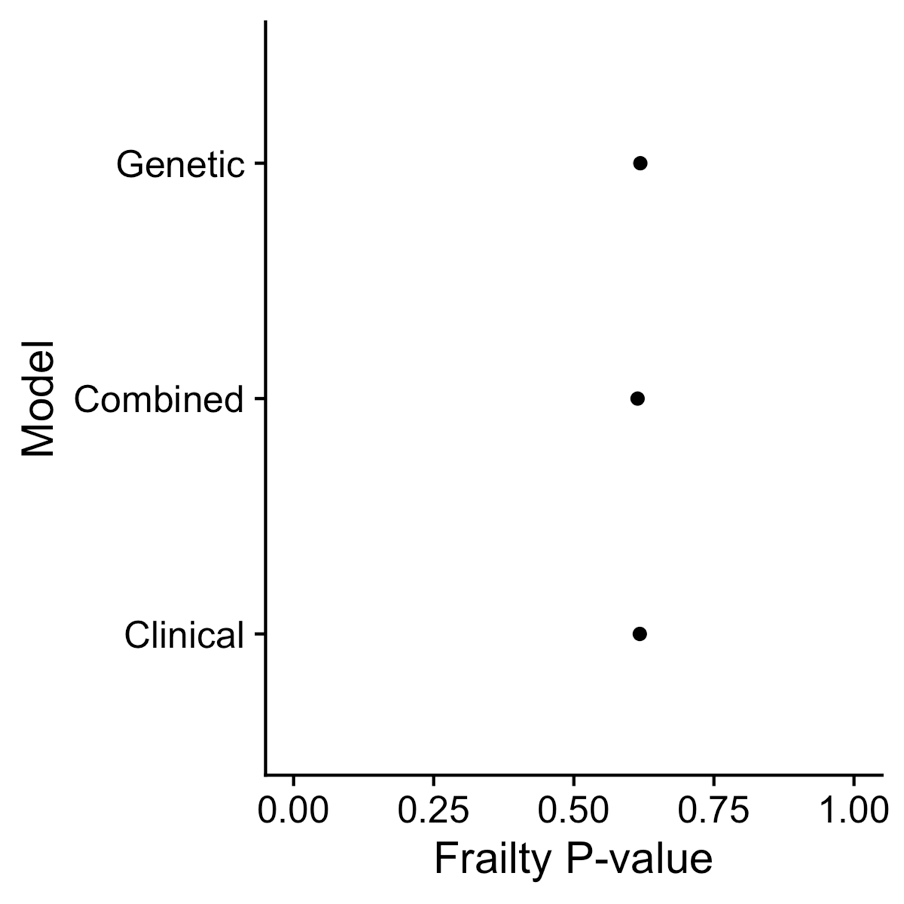


A frailty analysis was conducted to check for additional clustering among the participants. Three Fine-Gray models each including only the covariate listed on the y-axis were constructed.

**eFigure 18.** Schoenfeld Residuals for Venous Thromboembolism at Various Times


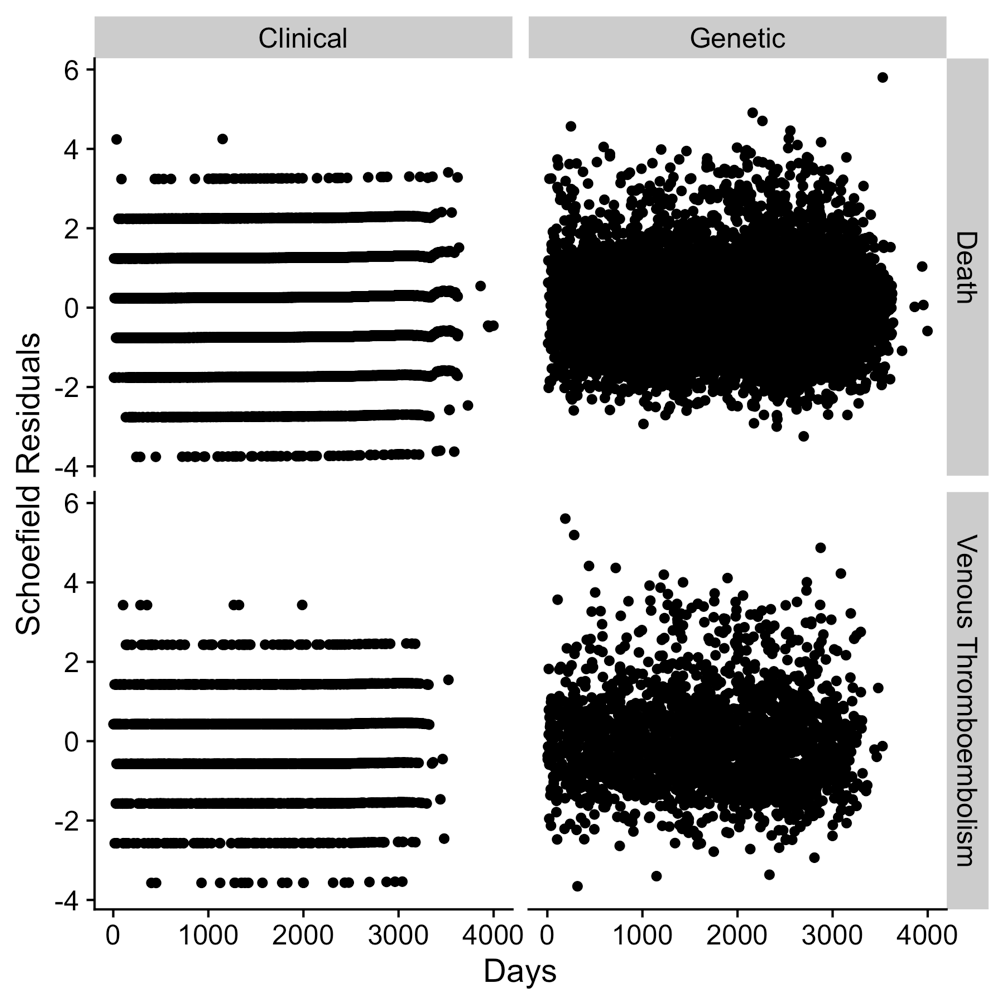


The Schoenfeld residuals were computed and plotted against the time course of the analysis to determine if there was any relationship between the time and either the genetic or clinical scores. To compute the Schoenfeld residuals, two Fine-Gray models, one for each of the competing risks, death and venous thromboembolism, were computed for both the clinical and genetic scores.

**eFigure 19.** Analysis of Fine-Gray Proportional Hazards Assumption


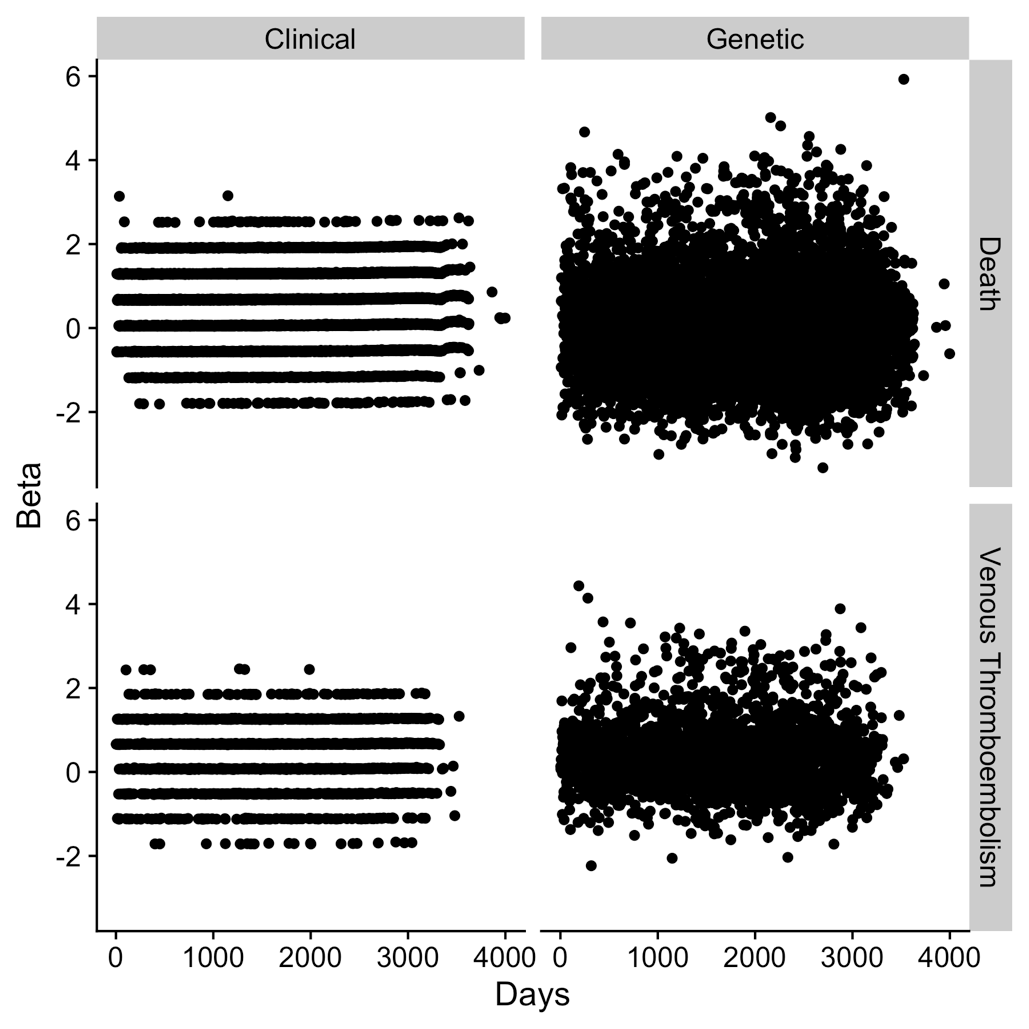


Shown is the analysis of the beta values for the clinical and genetic score values over time, to determine if the proportional hazard assumption is met. The beta values were generated with the cox.zph function from the survival package applied to two Fine-Gray models, one for each of the competing risks (death and venous thromboembolism), with both the clinical and genetic scores.

**eFigure 20.** Comparison of Fine-Gray and Cox Proportional Hazard Model Results


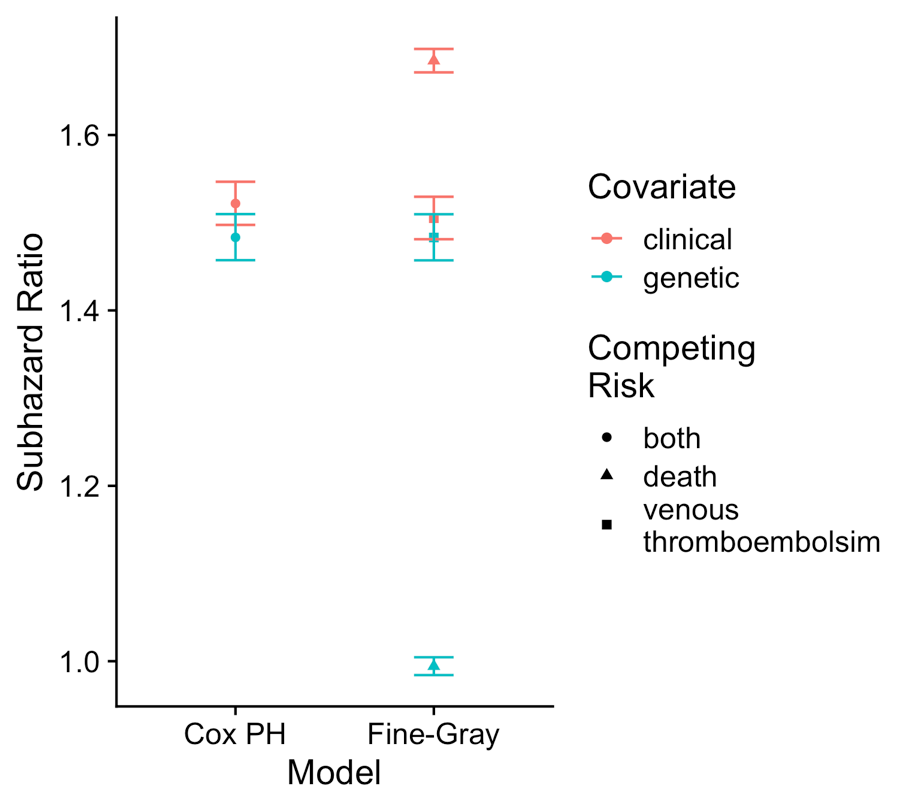


Here we show the comparison of the subhazard and hazard ratios generated from Fine-Gray and Cox proportional hazrd models for the simultaneous analysis of the genetic and clinical score. For these analyses, both venous thromboembolism and death were considered as separate outcomes. Error bars denote the standard error.

**eFigure 21.** Cumulative Incidence Curve of the Combined Score

**
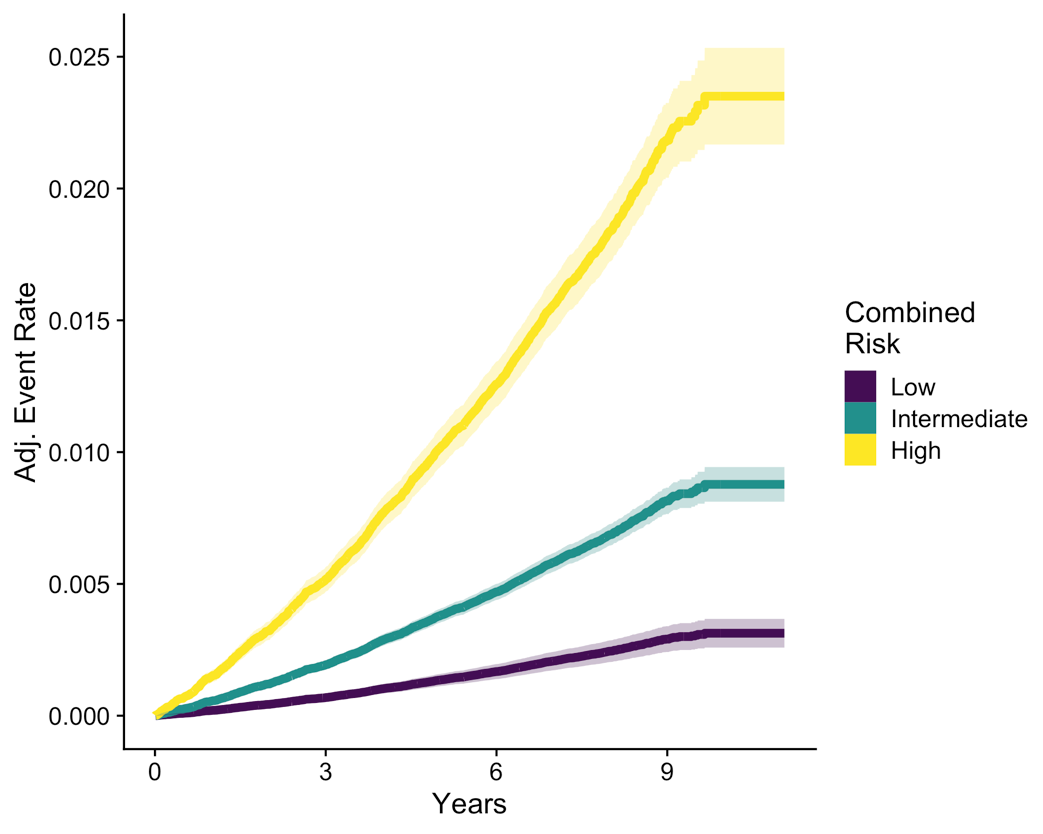
**

The cumulative incidences were generated with an unadjusted Fine-Gray model. The shaded regions represent the 95% confidence intervals.
